# Identifying emerging hot spots of road traffic injury severity using spatiotemporal methods: longitudinal analyses on major roads in Ghana from 2005 to 2020

**DOI:** 10.1186/s12889-024-18915-x

**Published:** 2024-06-17

**Authors:** Aldina Mesic, James Damsere-Derry, Caryl Feldacker, Stephen J. Mooney, Adam Gyedu, Charles Mock, Angela Kitali, Bradley H. Wagenaar, Daniel Hardy Wuaku, Martin Owusu Afram, Joshua Larley, Irene Opoku, Ernest Ekuban, Maxwell Osei-Ampofo, Barclay Stewart

**Affiliations:** 1 Department of Global Health, Hans Rosling Building, University of Washington, 3980 15th Avenue NE, Seattle, WA USA; 2 Building and Road Research Institute, Kumasi, Ghana; 3 Department of Epidemiology, University of Washington, Seattle, WA USA; 4 Harborview Injury Prevention and Research Center, Seattle, WA USA; 5 Department of Surgery, School of Medicine and Dentistry, Kwame Nkrumah University of Science and Technology, Kumasi, Ghana; 6 Department of Surgery, University of Washington, Seattle, WA USA; 7 Civil Engineering Program, University of Washington, Tacoma, Washington, USA; 8 National Road Safety Authority, Accra, Ghana; 9 Ghana Highway Authority, Accra, Ghana; 10 Department of Medicine, School of Medicine and Dentistry, Kwame Nkrumah University of Science and Technology, Kumasi, Ghana; 11 Directorate of Emergency Medicine, Komfo Anokye Teaching Hospital, Kumasi, Ghana; 12 Department of Primary Care and Public Health, Imperial College London, London, United Kingdom

**Keywords:** Road traffic injury, Spatial epidemiology, GIS, Injury prevention

## Abstract

**Background:**

Although road traffic injuries and deaths have decreased globally, there is substantial national and sub-national heterogeneity, particularly in low- and middle-income countries (LMICs). Ghana is one of few countries in Africa collecting comprehensive, spatially detailed data on motor vehicle collisions (MVCs). This data is a critical step towards improving roadway safety, as accurate and reliable information is essential for devising targeted countermeasures.

**Methods:**

Here, we analyze 16 years of police-report data using emerging hot spot analysis in ArcGIS to identify hot spots with trends of increasing injury severity (a weighted composite measure of MVCs, minor injuries, severe injuries, and deaths), and counts of injuries, severe injuries, and deaths along major roads in urban and rural areas of Ghana.

**Results:**

We find injury severity index sums and minor injury counts are significantly decreasing over time in Ghana while severe injury and death counts are not, indicating the latter should be the focus for road safety efforts. We identify new, consecutive, intensifying, and persistent hot spots on 2.65% of urban roads and 4.37% of rural roads. Hot spots are intensifying in terms of severity and frequency on major roads in rural areas.

**Conclusions:**

A few key road sections, particularly in rural areas, show elevated levels of road traffic injury severity, warranting targeted interventions. Our method for evaluating spatiotemporal trends in MVC, road traffic injuries, and deaths in a LMIC includes sufficient detail for replication and adaptation in other countries, which is useful for targeting countermeasures and tracking progress.

**Supplementary Information:**

The online version contains supplementary material available at 10.1186/s12889-024-18915-x.

## Background

Road traffic injuries are the leading cause of death among children and young adults 5 to 29 years of age [1]. The global incidence of road traffic injuries has increased while mortality has declined [2]. Increases in road traffic injuries are due to global increases in motorized transport and vulnerable road users (VRUs), whereas infrastructure, legislation, and improved post-crash care have led to decreases mortality [1, 2]. Despite global mortality reductions, there is substantial regional, national, and sub-national heterogeneity [2, 3]. An estimated 90% of deaths from road traffic injuries occur in low- and middle-income countries (LMICs) despite comparatively low numbers of motorized vehicles (an estimated 47% of the world’s vehicles) [4, 5].

Countries have committed to reducing road traffic injuries and deaths on roads through commitments such as the *United Nations (UN) Sustainable Development Goal (SDG) 3.6* and the adopted resolution for the *UN Decade of Action for Road Safety 2011–2020* and the second *Decade of Action for Road Safety 2021–2030*, all which aim to reduce road traffic injuries and deaths by 50% [6–8]. To understand whether countries are making progress toward achieving these ambitious targets, it is critical to monitor and use data for road safety planning and interventions.

Increasingly, LMICs are beginning to track detailed information on motor vehicle collisions (MVCs), road traffic injuries, and deaths to understand and address road safety issues, a common practice in most high-income countries [9]. Since 1991, Ghana has been one of the few African countries collecting comprehensive, spatially detailed data on MVCs, injuries, and deaths, which can drastically improve the ways in which changing patterns can be identified and used to inform decision-making, implementation, and evaluations. Specifically, spatial statistics can identify trends to allow for policymakers and public health practitioners to detect particularly high-risk areas and target injury prevention and control efforts. This is well aligned with a leading theory in road safety, the Safe Systems Approach, which focuses on eliminating serious road traffic injuries and fatalities by proactively and holistically addressing road system components [10, 11].

Like other LMICs, Ghana has a growing burden of road traffic injuries and deaths. The World Health Organization (WHO) estimated 8,494 deaths (25.9 deaths per 100,000 people) in 2021 [12]. The cost of severe road traffic injuries and deaths was an estimated 8.2% of the country’s Gross Domestic Product in 2016 [13]. In 2021, the total number of registered vehicles was 3,314,215, a 60% rise from the 2,066,943 vehicles recorded in 2018 [1, 12]. There has also been a surge in the number of powered two- and three-wheelers, which now make up an estimated 24.9% of the total vehicle fleet [14]. In the past two decades, the majority of injuries and deaths have been among vulnerable road users, with a notable increase in the proportion among occupants of powered two- and three-wheelers recently (2.7% of deaths in 2001 to 34% of deaths in 2021) [15]. Despite the substantial morbidity and mortality, Ghana does not comply to all UN conventions for national road safety legislation and has heterogenous implementation of the existing policies. National legislation that is absent includes laws requiring vehicles to have front and side protection, antilock braking, electronic stability control systems, and pedestrian protection, and a law mandating child restraints [12]. Although several other policies to address key risk factors (drink, drug, and distracted driving, helmets, seatbelts, speed, post-crash care) exist, there are documented variations in implementation and enforcement. For example, a recent study found that the level of overall compliance with road safety legislation among commercial motorcyclists was 59.2% [16]. Limited adherence has also been documented for seatbelts [17–19], helmets [20, 21], drink driving [22], and excessive speeding [23, 24].

Moreover, Ghana, like many other LMICs, did not reach the *UN Decade of Road Safety 2011–2020* goal to reduce road traffic injuries and deaths by 50% by 2020. Limited progress underscores the urgency in analyzing spatiotemporal trends, a practice commonly employed in high-resource settings [25]. When reviewing geospatial hotspot analyses studies in Africa, we found only 19 studies have been published since 2000, primarily in Nigeria [26–32] and Ethiopia [33–36]. These studies have some notable limitations, such as solely providing descriptive reports of the count or frequency of road outcomes [26, 31, 35, 37, 38], lacking spatial specificity (i.e., outcomes aggregated to a large geographic area) [26–30, 32, 34, 38], or focusing on a limited geographical scale (i.e., one city) [39]. To address this research gap, we conducted a rigorous hotspot analysis study to provide information on the specific 100-meter areas of high risk along major roads in Ghana from 2017 to 2020 (*publication forthcoming*). While hotspot analyses are valuable in identifying high risk areas, they often fall short in capturing temporal trends. Similarly, while many global, regional, and country-specific studies document trends over time, they often lack detailed geographic information which is critical for targeting resources effectively [1, 2, 5, 40–44] In contrast, spatiotemporal analyses can offer a more comprehensive understanding by identifying risk clusters and tracking changes in these clusters over time. By leveraging available data, Ghana has the potential to serve as an exemplar for other LMICs initiating similar data collection efforts and aiming to identify trends and geographic road safety priorities.

We set out to demonstrate a method for evaluating spatiotemporal trends in MVCs, road traffic injuries, and deaths in an LMIC, and to provide sufficient detail to facilitate the replication and adaptation of these methods for other countries/agencies. The method includes aggregating data into a space-time cube, and a hot spot analysis which combines Getis-Ord Gi* statistic and the Mann-Kendall test. To the best of our knowledge, only one study in a LMIC has employed this method to understand the evolution of pedestrian hotspots in Ahmedabad and Kolkata, India, identifying consecutive hotspots in the high economic activity city areas and new hotspots near areas undergoing road construction and infrastructure development [45]. This method has been used in high-income countries, including in Seoul for MVCs involving elderly people [46], Western Australia for heavy vehicle collisions [47], China for urban MVCs [48], and North Dakota for single-lane departure MVCs [49]. We used 16 years of data (from 2005 to 2020). We used an established road traffic injury severity index that assigns greater weights based on road traffic injury severity to identify particularly unsafe and high-risk road locations. We also mapped and assessed absolute counts of MVCs, minor injuries, severe injuries, and deaths as secondary outcomes. We hypothesized that there would be statistically significant clusters of road traffic injury severity indices with distinct spatiotemporal patterns that could inform future priorities.

## Methods

### Study area

The African continent has the highest road traffic death rate (26.6 per 100,000 people) worldwide and has seen increases in road traffic injuries and deaths in the last three decades [5]. We selected Ghana for this study as it is one of few countries in Africa collecting detailed and georeferenced data on each police reported MVC, road traffic injury, and death using recommended standardized definitions (i.e., anyone killed immediately or dies within 30 days of the MVC) [1, 50]. This study is from 2005 to 2020. Although we expected that COVID-19 restrictions would affect movement, traffic patterns, and the associated road safety outcomes in 2020, we decided to include this year as Ghana implemented few COVID-19 restrictions (a 21-day partial lockdown which required people to stay home) and limited these restrictions to four urban areas (Accra, Tema, Kasoa, and Kumasi). We did not observe any differences in road safety outcomes in 2020 compared to the prior years.

The geographical focus is on the major road network in Ghana, which includes national, inter-regional, and regional roadways (termed ‘trunk road network’ in Ghana, ‘interstate’ or ‘highway’ system in other countries). MVCs have been compiled electronically in the current format since 2005 (although the database started in 1991), and MVCs on the major road network were the ones for which geospatial information was obtained. We limited the analysis to any roads reporting more than 10 MVCs a year, as many small regional roads comprise a small portion (3.8%) of the total MVCs.

### Data and outcomes

Since 1991, the Ghana Building and Road Research Institute (BRRI) and Ghana Police Service collaborated to collect and maintain information on MVCs, road traffic injuries, and deaths in the National Road Traffic Accident Database for the National Road Safety Authority (NRSA). Trained staff from the Motor Traffic and Transport Unit of the Ghana Police Service are dispatched to each reported MVC and injury in a hospital to collect data using a standardized crash report form.

Each observation includes road and weather environment conditions (e.g., rain or clear, potholes), vehicle characteristics (e.g., type, ownership), driver information (e.g., drinking status, age), and outcome (i.e., MVC resulting in property damage, or minor road traffic injury, or severe road traffic injury [defined as requiring hospitalization], or death within 30 days of the MVC for each road user involved. We present the variables in the *Supplementary Materials.*

BRRI geospatial information is stored as a route number assigned by the Ghana Highways Authority (GHA), a km marker, and a landmark such as the townhall. We obtained the national road network shapefile from GHA. We also obtained complementary road network datasets from open sources including OpenStreetMap and the Humanitarian Data Exchange. We accessed the European Commission’s Joint Research Center’s Global Human Settlement Layer data to determine urban versus rural areas [51]. This dataset presents the degree of urbanization for each one km grid by combining population density from national censuses and built-up surfaces from satellite imagery.

We used a road traffic injury severity index for each time (year) and location as our primary outcome, presented in *Eq. 1.* There is no consensus on the best index to use in LMICs, or the best weighing approach for outcomes [52, 53]. On the first, there are several different approaches, including the widely used KABCO scale established by the Federal Highway Administration.

Given our focus on reducing morbidity and mortality, we employed an indexing system that assigns greater weights to worse collision severity, aligned with prior studies conducted in New South Wales and in the Adelaide metropolitan areas, and with our in-country collaborators’ views on appropriate weights [12, 13]. We included minor road traffic injury, severe road traffic injury, and death counts as secondary outcomes. Our decision to use a road traffic injury severity index is grounded in our theoretical viewpoint that we can achieve the greatest impact by focusing on severe injuries and deaths on the roads rather than trying to prevent all MVCs, as the Safe Systems Approach recommends [10, 11].

#### Equation 1 Injury severity index

$$\text{Injury severity index }\text{= }3 *\text{ }{X}_{1}+1.8 *\text{ }{X}_{2}+1.3 *\text{ }{X}_{3}+\text{ }{X}_{4}\text{ }$$

Where:

*X*_1_ = *count of deaths*

*X*_2_ = *count of severe injuries*

*X*_3_ = *count of not severe injuries*

*X*_4_ = *count of collisions resulting in property damage only*

### Data processing and statistical analysis

**Fig. 1:**
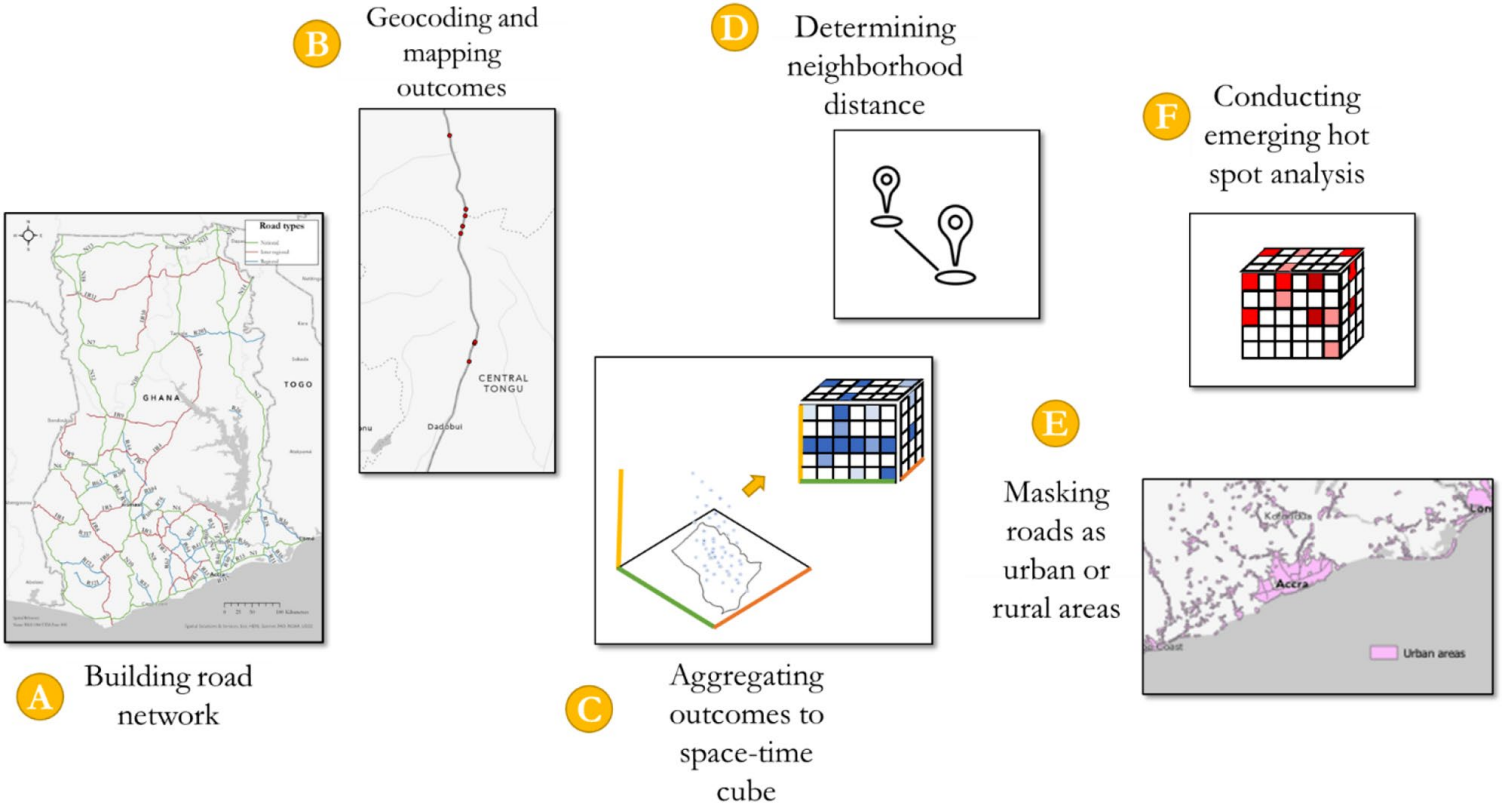
Data processing and statistical analysis workflow

We present a summary of the processing steps in Fig. 1. In the *Supplementary Materials*, we provide sufficient detail on data processing and statistical analysis decisions to improve replicability and scalability of the approach to other contexts.

First, we developed a road network by combining the GHA, Open Street Map, Humanitarian Data Exchange shape files, and BRRI’s strip maps. We consulted experts across the BRRI and GHA to verify the accuracy of the road network by checking the start point, end point, and curves along the road (A in Fig. 1). Next, we aggregated outcomes to each unique 100-meter location along the major road network. We then developed an automated process in ArcGIS Pro to calculate geocoordinates (X, Y) for a projected coordinate system relevant to Ghana (World Geodetic System 1984, Universal Transverse Mercator Zone 30 N). As part of this step, we quality checked the geocoordinates by comparing selecting a random subset of MVCs to manually map and verify using the BRRI strip maps (B in Fig. 1). Next, we transformed the data into a Network Common Data Form (netCDF) with a spatial resolution of 2 km using the Create Space Time Cube by Aggregating Points tool in ArcGIS Pro to sum all road traffic injury severity indices within each bin and period (C in Fig. 1). Our analysis included 16 years of data, the time slices, in the space-time cube. We aggregated data into 2 km bins by empirically testing different spatial resolutions. We selected this resolution to balance the needs of the research question (i.e., a larger area may not be relevant for a detailed understanding of the risk on roads) and the requirements of statistical tests (i.e., sufficient power and limited zeros for time series analysis). We present the results of a series of spatial resolutions in Table 1. Adjustments to bin size did not substantially affect results.

**Table 1:** Space-time cube spatial resolution

Outcome	Space-time cube resolution	Number of bins	% non-zero, sparseness
Road traffic injury severity index sum for each cube	500 m	1,605,990	24.92%
	1 km	402,420	31.72%
	2 km	50,689	40.64%
	2.5 km	44,807	43.78%
	5 km	16,330	53.91%

Then, we selected the neighborhood distances (D in Fig. 1) and overlaid the Global Human Settlement Layer data to determine which roads were in urban or rural areas for stratified analyses (E in Fig. 1). Lastly, we used the Emerging Hot Spot Analysis tool in ArcGIS and presented results in maps and figures (F in Fig. 1). Additional details on the neighborhood distances are provided below.

The Emerging Hot Spot Analysis tool is a space-time pattern mining tool that combines two statistical analyses: (1) the Getis-Ord Gi* statistic; and (2) the Mann-Kendall trend test for temporal trends. Input parameters for the tool include: (1) time step; (2) conceptualization of spatial relationships; and (3) polygon analysis mask, which are described in the *Supplementary Materials*. Briefly, we selected 3-year time steps, fixed distance band conceptualization, and to stratify by urban and rural areas by selecting differing distance for spatial relationships (e.g., 2 km for urban areas, 8 km for rural areas). There are 17 distinct categories of outcomes for the analysis, but we present only hot spots of road traffic injury severity and outcome counts that fall within four categories (new, consecutive, intensifying, persistent) defined in Table 2 below as the research team (in-country and global partners) decided these outcomes are the most relevant for understanding spatiotemporal patterns of road traffic injury. We have presented the results below and online in a Tableau Dashboard for decision-makers to access.

**Table 2:** Categories and definitions for emerging hot spot analysis

Pattern type	Definition
New hotspot	A location that is a statistically significant for the final time step (2018-2020) and has never been a statistically significant hotspot before.
Consecutive hotspot	A location with a single uninterrupted run of at least two statistically significant hotspot bins in the final time-step intervals (2015-2017 and 2018-2020). The location has never been a statistically significant hotspot before the final hotspot run, and less than 90% of all bins are statistically significant hotspots.
Intensifying hotspot	A location that has been a statistically significant hotspot for 90% of the time-step intervals, including the final time step (2018-2020). In addition, the intensity of clustering of high counts in each time step is increasing overall, which is statistically significant.
Persistent hotspot	A location that has been a statistically significant hotspot for 90% of the time-step intervals with no discernible trend in the intensity of clustering over time.

## Results

### Descriptive time trends

**Fig. 2:**
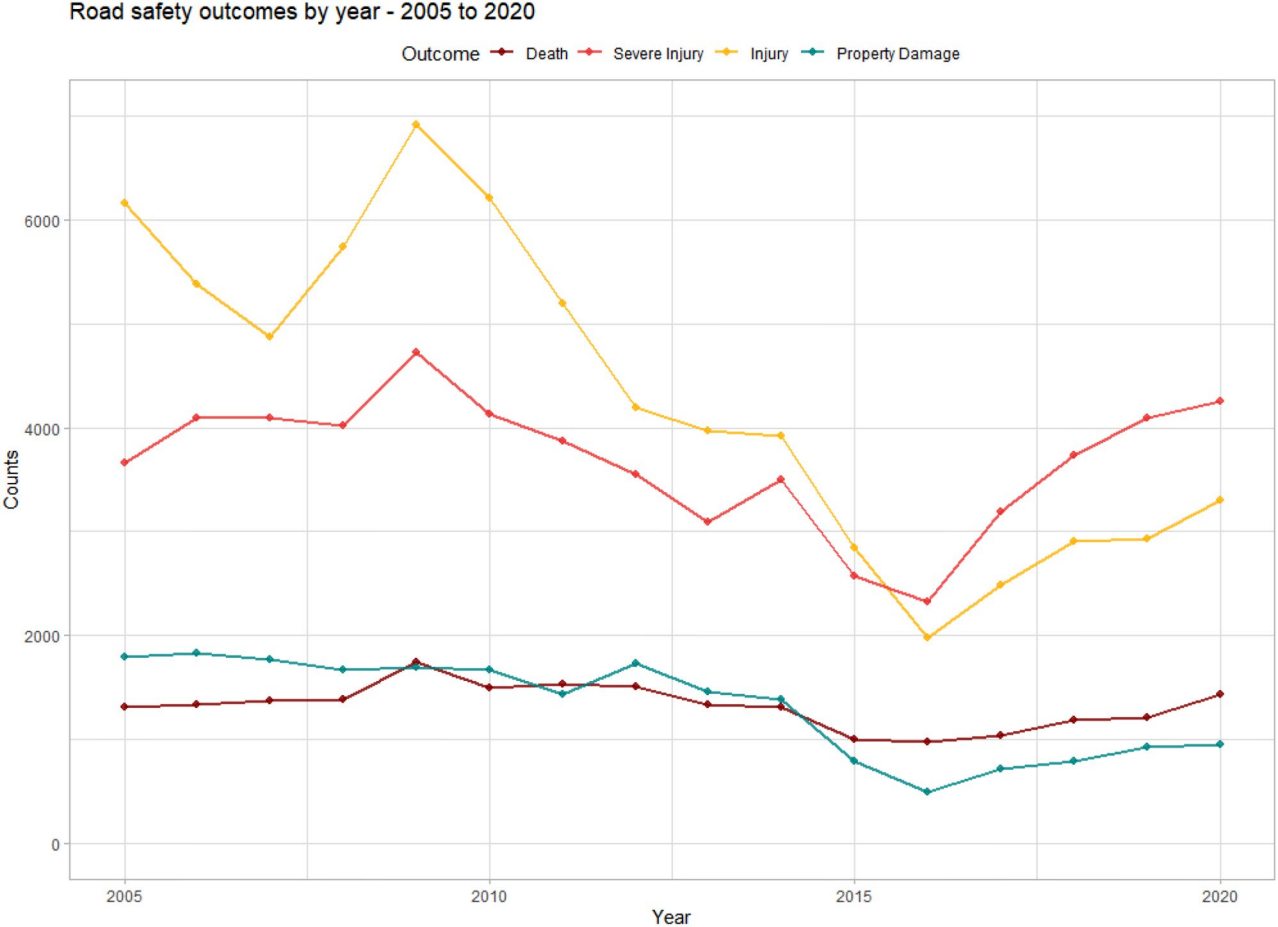
Annual sums of MVCs resulting in property damage, road traffic injuries, severe road traffic injuries, and deaths from 2005 to 2020

From 2005 to 2020, 87,441 police-reported MVCs occurred on major roads (59.3% of the 147,332 total MVCs) in Ghana. We limited the analysis to roads reporting more than 10 MVCs a year (84,118 of 87,441 MVCs; 96.2%), given that over 60 regional roads comprise less than 4% of the data. In total, BRRI reported 30,939 unique 100-meter locations along the major road network, with MVCs. Overall, all police-reported outcomes showed decreases from 2005 to 2015, with minor road traffic injuries and property damage decreasing most dramatically. Notably, property-damage MVCs and minor injuries are particularly prone to under-reporting, although we have no reason to believe that the reporting rate has changed over time. Upon observation, the sums of all police-reported outcomes seem to be increasing from 2015 to 2020. Since 2015, severe injury counts have surpassed all other outcomes (Table 3; Fig. 2).

**Table 3:** Yearly sums of MVCs resulting in property damage, road traffic injuries, severe road traffic injuries, and deaths (2005–2020)

**Outcome**	**2005**	**2006**	**2007**	**2008**	**2009**	**2010**	**2011**	**2012**	**2013**
Property damage	1,789	1,834	1,770	1,666	1,696	1,668	1,437	1,728	1,462
Minor road traffic injuries	6,158	5,387	4,870	5,734	6,919	6,210	5,193	4,191	3,976
Severe road traffic injuries	3,658	4,088	4,091	4,025	4,724	4,133	3,870	3,550	3,090
Deaths	1,312	1,328	1,370	1,380	1,739	1,491	1,527	1,506	1,338
road traffic injury severity mean*	5.08	5.51	5.43	5.30	5.87	5.85	5.68	5.68	5.13
**Outcome**	**2014**	**2015**	**2016**	**2017**	**2018**	**2019**	**2020**	**Total**	
Property damage	1,386	792	492	710	783	924	945	21,082	
Minor road traffic injuries	3,920	2,845	1,973	2,483	2,902	2,924	3,305	68,990	
Severe road traffic injuries	3,496	2,565	2,320	3,196	3,731	4,088	4,250	58,875	
Deaths	1,305	1,002	979	1,042	1,181	1,208	1,437	21,145	
road traffic injury severity mean*	5.44	5.18	5.62	5.30	5.71	5.88	5.75	5.52	

* For 100-meter locations reporting an MVC (*N* = 30,939 unique locations)

### Space-time cube results

There were 50,689 unique combinations of year and location (2 km by 2 km bins) of MVCs, minor road traffic injuries, severe road traffic injuries, and deaths across the 16 years with neighborhood distances of 2 km for urban areas and 8 km for rural areas. In the space-time cube, the overall road traffic injury severity index sum and minor injury counts trends decreased significantly (*P* = 0.0060 and *P* = 0.0014, respectively). However, severe road traffic injury and death counts did not (*P* = 0.3214 and *P* = 0.2241, respectively) (Table 4).

**Table 4:** Overall trends in data (2005–2020) in space-time cube

Outcome	Overall data trend of outcome (*P*-value)
Road traffic injury severity sum^A^	Decreasing (*P* = 0.0060)
Road traffic injury count^B^	Decreasing (*P* = 0.0014)
Severe road traffic injury count^B^	Not significant (*P* = 0.3214)
Death count^B^	Not significant (*P* = 0.2241)

The road traffic injury severity sums of each 2 km by 2 km bin. For example, if a bin has 2 MVCs in a 3-year period, one with a road traffic injury severity of 5, another with a road traffic injury severity of 100, then the sum would be 105 for that period/bin combination. This is the value used to assess time trends.

This is the total count of the outcome in each period and bin. For example, if a 2 km by 2 km bin has 5 MVCs in a period, all resulting in deaths, the death count would be 5. This is the value used to assess time trends.

### Emerging hot spot (spatio-temporal) results

**Fig. 3:**
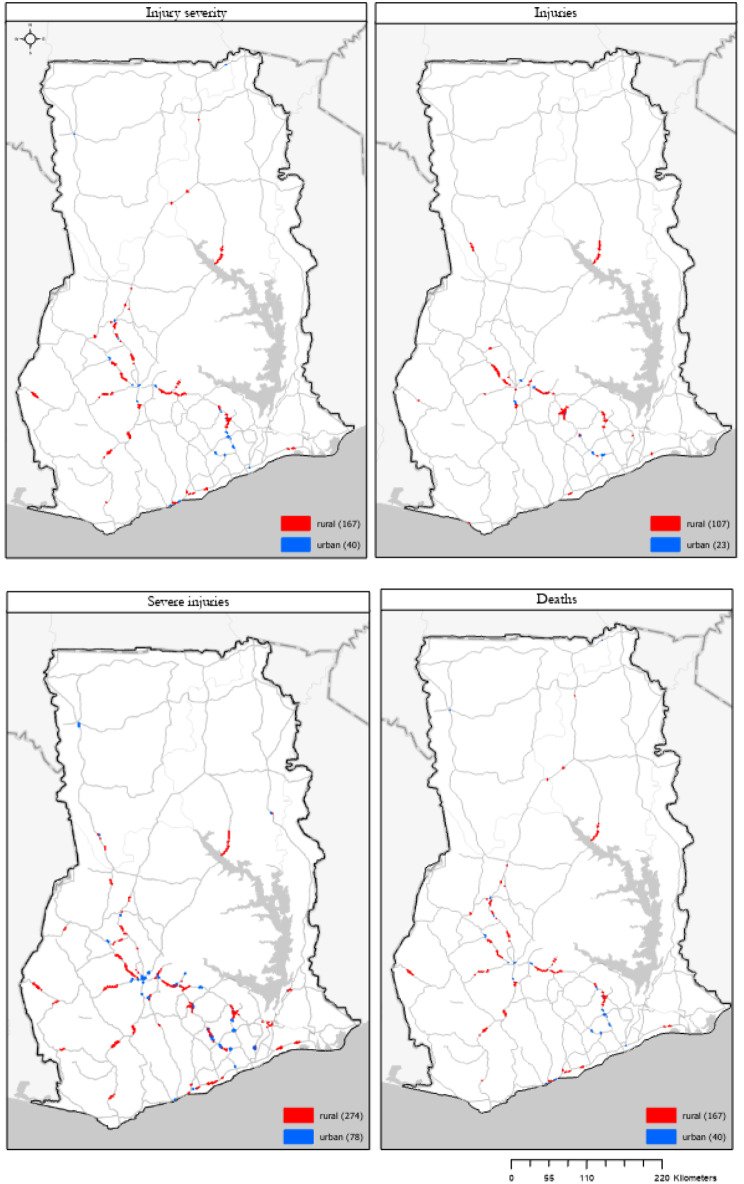
Emerging hot spot analysis (2005–2020) for the injury severity index, minor injuries, severe injuries, and deaths along major roads in urban and rural areas

We identified a small number of high-risk clusters along major roads in urban and rural areas (shown below in Table 5; Fig. 3). All clusters for all years are presented in a Tableau Dashboard. Severe injuries have the most clusters, followed by injury severity index clusters (Fig. 3*)*. Locations and patterns are broadly similar across outcomes. The hot spots of severe injuries and the injury severity index demonstrate nearly identical spatiotemporal patterns.

**Table 5:** Emerging hot spot results (2005–2020) along major roads in urban and rural areas summary

		Road traffic injury severity index	Road traffic injury counts	Severe road traffic injury counts	Death counts
Type of area	Category	N (%)	N (%)	N (%)	N (%)
Urban (*N* = 1,997 bins)	No cold or hot spot trends	1,850 (92.64%)	1,912 (95.74%)	1,810 (90.64%)	1,907 (95.49%)
	New hot spot	6 (0.30%)	6 (0.30%)	19 (0.95%)	13 (0.65%)
	Consecutive hot spot	37 (1.85%)	17 (0.85%)	53 (2.65%)	24 (1.20%)
	Intensifying hot spot	3 (0.15%)	0 (0%)	1 (0.050%)	0 (0%)
	Persistent hot spot	7 (0.35%)	0 (0%)	5 (0.25%)	3 (0.15%)
Rural (*N* = 4,299 bins)	No cold or hot spot trends	3,788 (88.11%)	4,058 (94.39%)	3,762 (87.51%)	3,925 (91.3%)
	New hot spot	35 (0.81%)	14 (32.56%)	61 (1.42%)	45 (1.04%)
	Consecutive hot spot	63 (1.47%)	28 (65.1%)	142 (3.30%)	66 (1.53%)
	Intensifying hot spot	29 (0.67%)	4 (0.09%)	19 (0.44%)	8 (0.18%)
	Persistent hot spot	96 (2.23%)	61 (1.41%)	52 (1.21%)	48 (1.12%)

When assessing the road traffic injury severity index sums, most locations (87.51–95.75%) across all outcomes and area types did not have a statically significant trend. We found three intensifying hot spots in urban areas and 29 (0.67%) in rural areas. Two urban clusters are in Kumasi, whereas another is located just north of Suhum in the Eastern Region. Several intensifying rural hot spots are found in Ejisu-Juaben District along National Road 6 and Regional Road 104 in the Ashanti region and on National Road 6 and Regional Road 32 in East Akim District in the Eastern Region (map in the *Supplementary Materials)*. These outcomes are particularly concerning given the MVCs are statistically more severe over time in these locations.

We found six new hot spots (0.30% of total area) in the urban areas and 35 (0.81%) in rural areas. The new urban hot spots were found in Kumasi city, east of Konongo in Asante Akim Central District, and in Mamangso in Birim North District. New rural hot spots were found all over the country but were often concentrated in a specific area, such as seven new hot spots on Inter-regional Road 5 between Kumasi and Bibiani in Atwima Mponua District.

We found 37 (1.87%) consecutive hot spots in urban areas and 63 (1.47%) in rural areas. Consecutive urban hot spots were all found on roads linking or between Kumasi and Accra, such as National Road 6 and Regional Road 64 in Central, Eastern, and Ashanti Regions. Rural consecutive hot spots were more widespread but followed similar patterns to the other categories (i.e., several concentrated along a specific stretch of road). Lastly, we observed seven (0.35%) persistent in urban areas and 96 (2.23%) in rural areas.

**Fig. 4:**
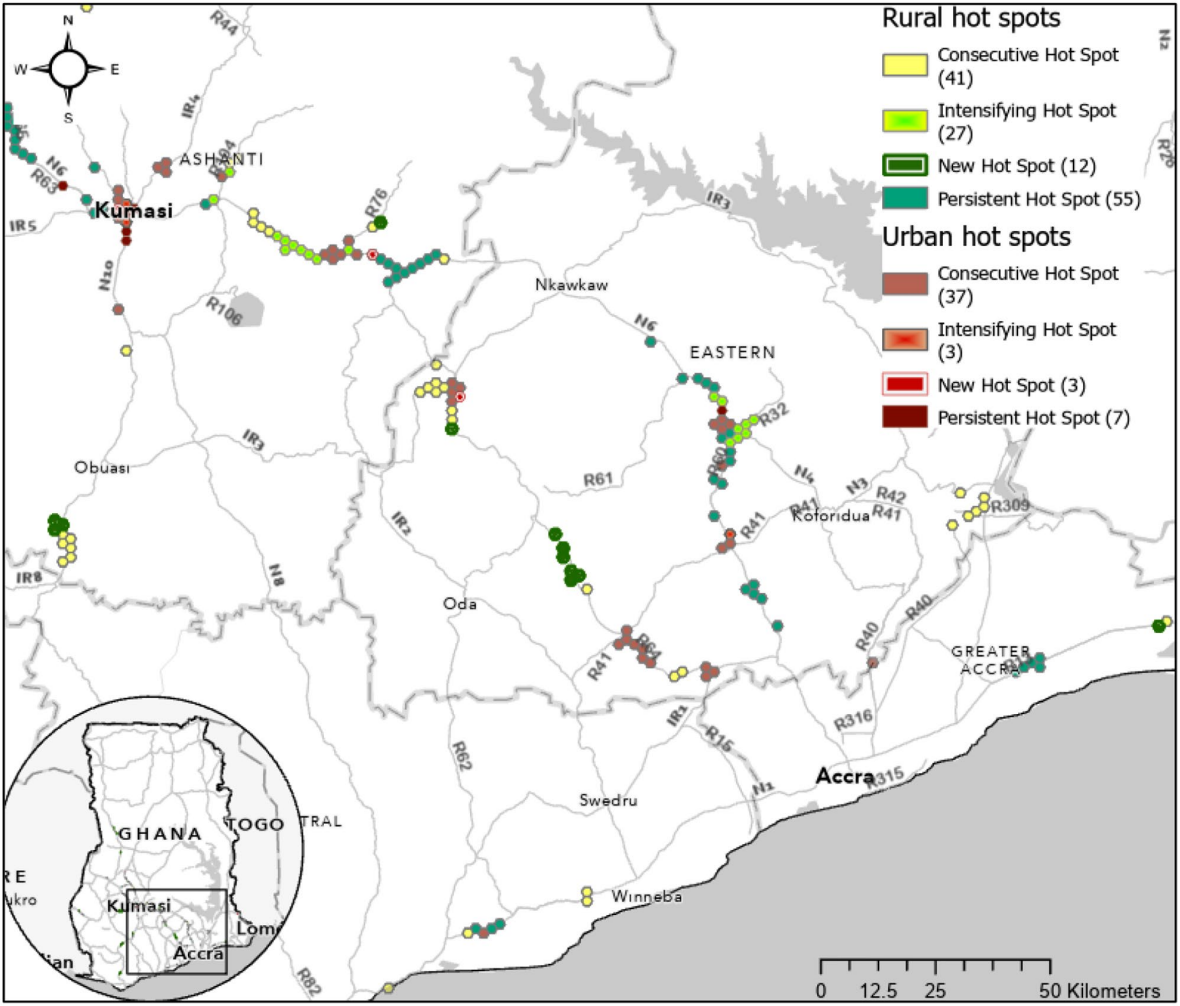
Key regions of emerging hot spots (2005–2020) along major roads in urban and rural areas

**Fig. 5:**
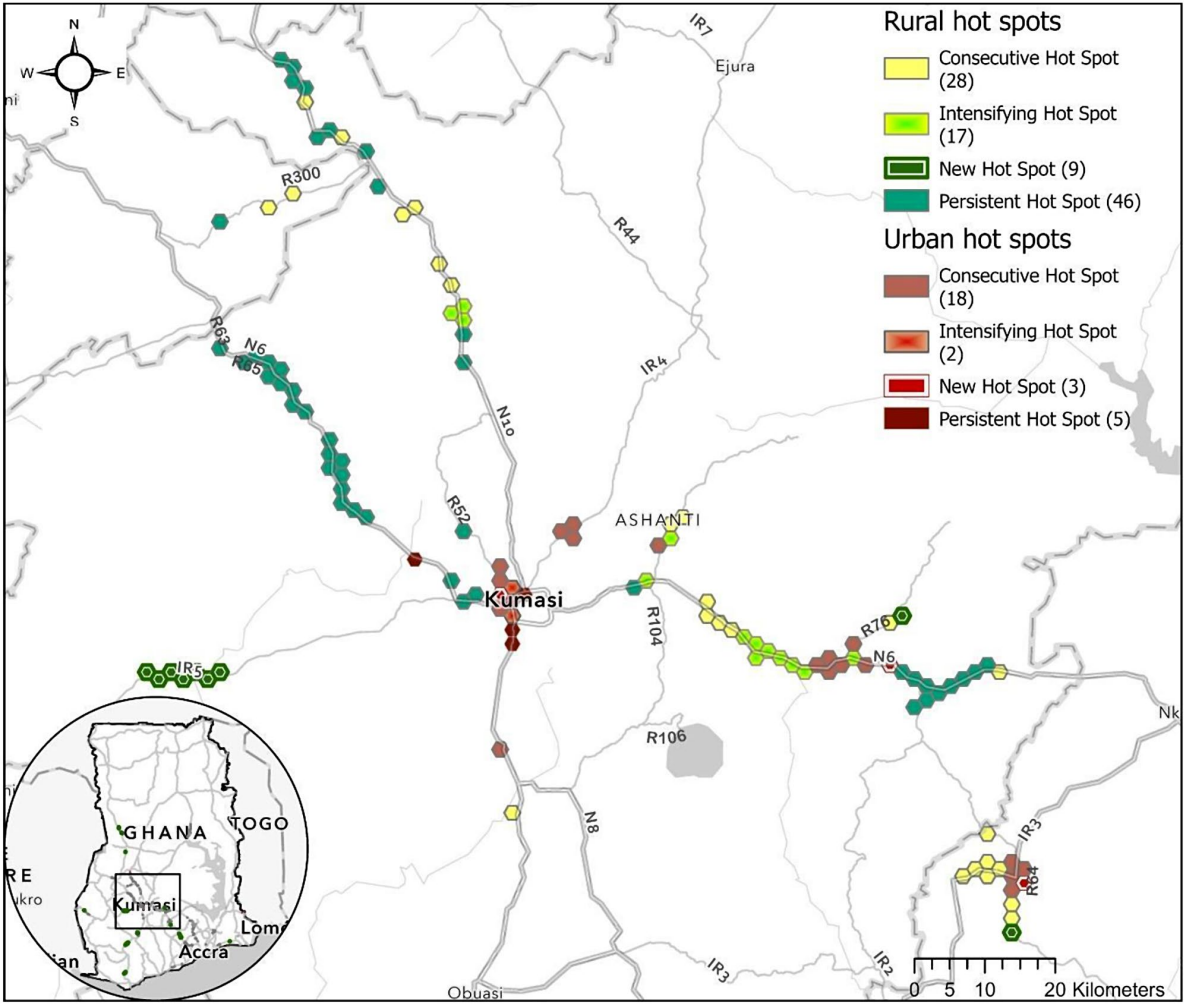
Emerging hot spots along major roads in urban and rural areas of Greater Kumasi

In Fig. 4, we highlight a few regions with significant patterns of road traffic injury severity over the 16-year period in Greater Accra and Eastern Region between three of Ghana’s largest cities (Accra, Kumasi, Cape Coast). To further demonstrate the spatial specificity and utility of this analysis and output for decision-making, we present a high-resolution map of greater Kumasi in Fig. 5. In this map, we have identified critical locations and information for decision-makers in the Ashanti region regarding which roads and stretches of roads are responsible for high injury severity in the country over 16 years. For example, the center of Kumasi has new, intensifying, and consecutive hot spots relative to the rest of the urban areas in Ghana, indicating it is of high priority to address. National Road 6, southeast and northwest of Kumasi, show persistent and intensifying trends of hot spots. National Road 6, and Regional Roads 64 and 32 have several concentrated areas of high risk.

## Discussion

We assessed police-reported road traffic injury patterns over space and time in Ghana from 2005 to 2020. We find the road traffic injury severity index and minor injuries are decreasing significantly; however, severe injuries and deaths are on the rise indicating that increasing counts of severe injuries and deaths should be the focus of future road safety efforts, a finding which aligns with the Safe Systems Approach [10, 11]. Observationally, we find that all outcomes show reductions up until 2015, which may be attributed to the government’s road safety efforts.vehicle composition and traffic patterns. Since 2016, all outcomes have shown increases and severe injuries have surpassed all other outcomes. The rapid expansion of all vehicles, and particularly motorized two- and three-wheelers (15,136 newly registered in 2005 compared to 105,059 in 2020) may explain this trend. These vehicles have altered road traffic injury trends, exacerbating the problem for VRUs (e.g., pedestrians, cyclists, and occupants of powered two-and three-wheelers) [54].

Our analysis identified a small proportion of the major roads to target for injury prevention (2.65% of major roads in urban areas; and 4.37% of major roads in urban areas) to shift alarming trends. Specifically, we identified three specific 2 km by 2 km urban areas and 29 specific 2 km by 2 km rural areas, which are becoming more severe hot spots over time (“intensifying”). By using a data-driven approach which focuses on 7% of the major roads, we transform a national problem of high injuries and deaths into a more manageable and actionable issue. This highly targeted approach enables a more efficient and potentially effective deployment of scarce road safety resources, one which is needed to meet the goals of the second *Decade of Action for Road Safety 2021–2030.*

Trends in rural areas are particularly concerning. Studies have documented the persistent issue of road traffic injuries and deaths in these areas for decades [44, 55], and we now have data to support the issue’s urgency. Understanding related factors, such as land use changes, road degradation, or disparities in post-crash care, is crucial. The notable differing trends in rural and urban areas support urbanization-stratified study designs, such as a recent analysis of the predictors of injury severity among motorcyclists in urban areas versus rural areas [56]. Understanding and altering these temporal trends on a granular level may change the overall trajectory of road safety outcomes and progress toward national and international targets.

In line with this, we have two key recommendations for the Ghanaian government, implementers, and researchers. First, the government should examine and act on the areas we have outlined for injury prevention with a specific focus on the rural hot spots, which have increased in frequency and severity. Secondly, we recommend follow up analyses include a detailed stratified analysis of characteristics for MVCs to better understand the key drivers in the specific hot spot locations, and in these areas overall. Beyond this, a road user-specific (e.g., pedestrians) or vehicle-specific (e.g., motorcycle, motorized tricycle) analysis would provide important details on what is causing the spatiotemporal patterns we observe.

Beyond addressing existing issues, this method contributes to predicting future trends, a key distinction from related hot spot analyses. Hot spot analyses and similar spatial assessments retrospectively identify areas of high risk. This work goes beyond that by capturing both the spatial and temporal dimensions, enabling a more nuanced understanding of which areas or road users are most affected and may be experiencing deteriorating safety conditions (i.e., the intensifying hot spots). Advancements in technology, computing, and data (e.g., real-time electronic crash reports) could enable this method to be used frequently in real time, rather than as a periodic review of trends.

In this report, we applied spatiotemporal analyses to Ghana, but we have outlined our methods, model assumptions, and analytic decisions with sufficient detail to facilitate research for road safety practitioners in other countries. This method has been applied to understand pedestrian hotspots in India [45], and for several MVC analyses in high-income settings [46–49]. To the best of our knowledge, this is one of the first attempts to understand emerging trends and geographic priorities nationally in Africa. It also underscores the importance of frequently collecting and monitoring road traffic injury and death data on a granular level, so that we can move beyond simple descriptions and identify emerging trends to guide future interventions.

For other countries in Africa and beyond that may consider similar analyses, we outline some key components. First, georeferenced data on MVCs is required. This is a notable gap in LMICs, while the benefits of collecting and maintaining these data cannot be overstated given the disproportionate burden of road traffic injuries and deaths in these settings [50]. Next, the data must also have at least 10-time steps (e.g., 10 years, months, or weeks of data depending on the timescale of interest). Finally, given how crucial the key parameter selection is (e.g., neighborhood distance, bin size), each aspect should be discussed and decided with in-country partners to ensure context-relevance.

This study has some limitations to note. We rely on police-reported data on MVCs, minor road traffic injuries, severe road traffic injuries, and deaths, which may be underreported, particularly for less severe outcomes. There is a notable difference between the BRRI and WHO estimates for outcomes (e.g., over 8,000 annual deaths in the WHO estimates in 2021). This discrepancy is not unique to Ghana [57–59]. We have no reason to believe that this data is missing in a non-systematic way over the study period, as the data collection methodology was consistent. As such, we believe that this data is useful, particularly for evaluating trends over time. Closely related, we did not apply a correction factor due to limited evidence on appropriate factor to use for each outcome [60]. Second, our dataset includes 2020, when the COVID-19 pandemic occurred, which may have influenced road safety outcomes. However, in the aggregate, 2020 did not seem dramatically different from other years given limited COVID-19 measures. Next, we do not include exposure data (e.g., vehicle counts), as this is not available in Ghana or many other LMICs. Next, many test specifications (e.g., bin size, and neighborhood distance) are subjective. We selected these parameters by considering the research question and context to design an analysis that could arm policymakers with meaningful and specific findings for future road safety efforts. Finally, we opted to use a weighted index for injury severity, which assigns more severe outcomes greater weights. We also conducted the analysis for outcome counts to account for potential disagreements about the weighting decisions.

Despite these limitations, our study demonstrates how the increasing availability of georeferenced road traffic injury and death data allows for analyzing and monitoring spatiotemporal trends. Trends can inform decision-making related to injury prevention and track progress toward national and international goals, forecast future patterns, and increase equity in addressing these global challenges.

### Electronic supplementary material

Below is the link to the electronic supplementary material.

Supplementary Material 1

## Data Availability

The Building and Road Research crash database cannot be accessed publicly. We sought permission to access the dataset for this study. To facilitate dissemination, we have included the results on a public Tableau dashboard at the following link: https://public.tableau.com/app/profile/aldina.mesic/viz/Spatio-temporaltrendsinroadtrafficinjuryinGhana2005-2020/Sheet1?publish=yes.

## References

[1] Global status report on road safety 2018. (2018). Geneva, Switzerland, WHO. 2019.

[2] James SL, Lucchesi LR, Bisignano C, Castle CD, Dingels ZV, Fox JT (2020). Morbidity and mortality from road injuries: results from the global burden of Disease Study 2017. Inj Prev.

[3] Nantulya VM, Reich MR (2003). Equity dimensions of road traffic injuries in low-and middle-income countries. Injury Control Saf Promotion.

[4] Harikrishnan S, Jeemon P, Mini G, Thankappan K, Sylaja P. GBD 2017 causes of death collaborators. Global, regional, and national age-sex-specific mortality for 282 causes of death in 195 countries and territories, 1980–2017: a systematic analysis for the global burden of Disease Study 2017. 2018. 10.1016/S0140-6736(18)32203-7 PMC6227606 30496103

[5] Organization WH. Global status report on road safety 2013: supporting a decade of action: summary. World Health Organization; 2013.

[6] Nations U. Sustainable Development Goals [ https://sdgs.un.org/goals.

[7] Krug E (2012). Decade of action for road safety 2011–2020. Injury.

[8] Organization WH. Global plan for the decade of action for road safety 2021–2030. WHO Regional Office for the Western Pacific; 2022.

[9] Huang H, Yin Q, Schwebel DC, Ning P, Hu G (2017). Availability and consistency of health and non-health data for road traffic fatality: analysis of data from 195 countries, 1985–2013. Accid Anal Prev.

[10] Mooren L, Grzebieta R, Job R, Williamson A, editors. Safe System–International Comparisons of this Approach. A Safe System-making it happen: Proceedings of the Australasian College of road Safety Conference, Melbourne; 2011.

[11] Belin M-A (2016). Vision zero as a new way of thinking. J Australasian Coll Road Saf.

[12] Organization WH. Road safety Ghana 2023 country profile. 2024.

[13] Ghana’s Road Safety Country Profile. Global Road Safety Facility 2022 [ https://www.roadsafetyfacility.org/country/ghana.

[14] Africa S-S. THE WHEELS OF CHANGE.

[15] Akweley Issaah M. Motorcycle fatalities constitute 34 per cent of Ghana’s road traffic crashes in 2021. Ghana News Agency. 2022.

[16] Hagan D, Tarkang EE, Aku FY (2021). Compliance of commercial motorcycle riders with road safety regulations in a peri-urban town of Ghana. PLoS ONE.

[17] Okyere P, Agyei-Baffour P, Harris MJ, Mock C, Yankson IK, Donkor P (2021). Barriers to the enforcement of mandatory seat belt laws in Ghana: an exploratory study. Health Promot Int.

[18] Densu SN, Salifu M (2013). Occupant protection: observed seatbelt use in the Sekondi-Takoradi Metropolis (STM), Ghana. Int J Struct Civil Eng Res.

[19] Agyemang E (2018). Towards reducing the dangers associated with road traffic accidents: seat belt use and explanatory factors in the Accra metropolis of Ghana. Ghana J Geogr.

[20] Ackaah W, Afukaar FK (2010). Prevalence of helmet use among motorcycle users in Tamale Metropolis, Ghana: an observational study. Traffic Inj Prev.

[21] Nimako Aidoo E, Bawa S, Amoako-Yirenkyi C (2018). Prevalence rate of helmet use among motorcycle riders in Kumasi. Ghana Traffic Injury Prev.

[22] Damsere-Derry J, Palk G, King M (2017). Motorists’ knowledge, attitudes and practices toward alcohol-impaired driving/riding in Ghana. Traffic Inj Prev.

[23] Derry JD, Afukaar FK, Donkor P, Mock C (2007). Study of vehicle speeds on a major highway in Ghana: implication for monitoring and control. Traffic Inj Prev.

[24] Damsere-Derry J, Afukaar FK, Donkor P, Mock C (2008). Assessment of vehicle speeds on different categories of roadways in Ghana. Int J Injury Control Saf Promotion.

[25] Aziz S, Ram S (2022). A Meta-analysis of the methodologies practiced worldwide for the identification of Road Accident Black spots. Transp Res Procedia.

[26] Aderamo A (2012). Spatial pattern of road traffic accident casualties in Nigeria. Mediterranean J Social Sci.

[27] Rukewe A, Taiwo O, Fatiregun A, Afuwape O, Alonge T, GEOGRAPIC, INFORMATION SYSTEMS IN DETERMINING ROAD TRAFFIC CRASH ANALYSIS IN IBADAN (2014). NIGERIA J West Afr Coll Surg.

[28] Korter GO, Olubusoye OE, Salisu MA (2013). Spatio-temporal analysis of characteristics and causes of road traffic crashes in Oyo state of Nigeria. Nigerian Health J.

[29] Daniel OJ, Adejumo OA, Oritogun KS, Mautin GJ, Salako AA (2017). Spatial epidemiology of Road Traffic crashes and Mortality in Nigeria, 2007–2015. Br J Appl Sci Technol Terry J Ellapen South Afr Nuwadatta Subedi Gandaki Med Coll.

[30] Akinyemi YC (2019). Exploratory spatial analysis of traffic crashes, road mortality and morbidity in Nigeria. Int Social Sci J.

[31] Yahaya AM, Yusuf AS, Musa I, Maaji FI, Bambale SU, Oscar R et al. A spatiotemporal appraisal of road traffic accident in Kaduna metropolis, Nigeria.

[32] Audu AA, Iyiola OF, Popoola AA, Adeleye BM, Medayese S, Mosima C (2021). The application of geographic information system as an intelligent system towards emergency responses in road traffic accident in Ibadan. J Transp Supply Chain Manage.

[33] AkliluToma S, Senbeta BA, Bezabih AA (2021). Spatial distribution of Road Traffic Accident at Hawassa City Administration, Ethiopia. Ethiop J Health Sci.

[34] Tola AM, Demissie TA, Saathoff F, Gebissa A (2021). Severity, spatial pattern and statistical analysis of road traffic crash hot spots in Ethiopia. Appl Sci.

[35] Hayidso TH, Gemeda DO, Abraham AM (2019). Identifying road traffic accidents hotspots areas using GIS in Ethiopia: a case study of Hosanna Town. Transp Telecommunication.

[36] Tola AM, Gebissa A (2019). Identifying Black Spot Accident zones using a geographical information system on Kombolcha-Dessie Road in Ethiopia. Int J Sci Basic Appl Res.

[37] BakamaNume BB (2006). Road traffic accidents in Uganda: epidemiological and transport policy implications. Afr Social Sci Rev.

[38] Atubi A (2012). Epidemiology of injuries from road traffic accidents in Lagos State. Nigeria AFRREV STECH: Int J Sci Technol.

[39] Patel A, Krebs E, Andrade L, Rulisa S, Vissoci JRN, Staton CA (2016). The epidemiology of road traffic injury hotspots in Kigali, Rwanda from police data. BMC Public Health.

[40] Violence WHO, Prevention I, Organization WH. Global status report on road safety: time for action. World Health Organization; 2009.

[41] Organization WH. Global status report on road safety 2023 2023.

[42] Siaw NA, Duodu E, Sarkodie K (2013). Trends in road traffic accidents in Ghana; implications for improving road user safety. Int J Humanit Social Sci Invention.

[43] Twenefour F, Ayitey E, Kangah J, Brew L. Time series analysis of road traffic accidents in Ghana. Asian J Probab Stat. 2021.

[44] Afukaar FK, Antwi P, Ofosu-Amaah S (2003). Pattern of road traffic injuries in Ghana: implications for control. Injury Control Saf Promotion.

[45] Hussain MS, Goswami AK, Gupta A. Predicting pedestrian crash locations in urban India: an integrated GIS-based spatiotemporal HSID technique. J Transp Saf Secur. 2022:1–34.

[46] Kang Y, Cho N, Son S (2018). Spatiotemporal characteristics of elderly population’s traffic accidents in Seoul using space-time cube and space-time kernel density estimation. PLoS ONE.

[47] Gudes O, Varhol R, Sun QC, Meuleners L (2017). Investigating articulated heavy-vehicle crashes in Western Australia using a spatial approach. Accid Anal Prev.

[48] Wu P, Meng X, Song L (2022). Identification and spatiotemporal evolution analysis of high-risk crash spots in urban roads at the microzone-level: using the space-time cube method. J Transp Saf Secur.

[49] Khan IU, Vachal K, Ebrahimi S, Wadhwa SS. Hotspot analysis of single-vehicle lane departure crashes in North Dakota. IATSS Res. 2022.

[50] Segui Gomez M, Addo-Ashong T, Raffo VI, Venter P. Road Safety Data In Africa. 2021.

[51] GHSL - Global Human Settlement Layer. In. Nations U, editor 2022.

[52] Geurts K, Wets G, Brijs T, Vanhoof K (2004). Identification and ranking of black spots: sensitivity analysis. Transp Res Rec.

[53] Billah K, Sharif HO, Dessouky S (2023). Bivariate-logit-based severity analysis for Motorcycle crashes in Texas, 2017–2021. Sustainability.

[54] Aidoo EN, Amoh-Gyimah R (2020). Modelling the risk factors for injury severity in motorcycle users in Ghana. J Public Health.

[55] Bawah A, Welaga P, Azongo DK, Wak G, Phillips JF, Oduro A (2014). Road traffic fatalities-a neglected epidemic in rural northern Ghana: evidence from the Navrongo demographic surveillance system. Injury Epidemiol.

[56] Agyemang W, Adanu EK, Jones S (2021). Understanding the factors that are associated with motorcycle crash severity in rural and urban areas of Ghana. J Adv Transp.

[57] Elvik R, Mysen A (1999). Incomplete accident reporting: meta-analysis of studies made in 13 countries. Transp Res Rec.

[58] Samuel JC, Sankhulani E, Qureshi JS, Baloyi P, Thupi C, Lee CN (2012). Under-reporting of road traffic mortality in developing countries: application of a capture-recapture statistical model to refine mortality estimates. PLoS ONE.

[59] Watson A, Watson B, Vallmuur K (2015). Estimating under-reporting of road crash injuries to police using multiple linked data collections. Accid Anal Prev.

[60] Salifu M, Ackaah W (2012). Under-reporting of road traffic crash data in Ghana. Int J Injury Control Saf Promotion.

