# Supplementary material for: Identifying emerging hot spots of road traffic injury severity using spatiotemporal methods: longitudinal analyses on major roads in Ghana from 2005 to 2020
Source: BMC Public Health. 2024 Jun 17;24:1609. doi: 10.1186/s12889-024-18915-x (PMC11181649; doi:10.1186/s12889-024-18915-x)

**Supplementary Materials**

Detailed statistical analysis

The Emerging Hot Spot Analysis is a space-time pattern mining tool that combines two statistical analyses: 1) the Getis-Ord Gi* statistic; and 2) the Mann-Kendall trend test for temporal trends. The Getis-Ord Gi* method identifies statistically significant spatial clusters (Getis and Ord 2010). Results of the analysis are presented as Z scores, which indicate high/low values of bins (in this case, the sum of road traffic injury severity indices and counts of minor road traffic injuries, severe road traffic injuries, and deaths in each bin) that are near each other. This tool considers the Z value of the bin, the Z values of surrounding bins, and the confidence levels (90%, 95%, and 99%). Large, positive Z values indicate high levels of clustering (i.e., a hotspot meaning bins with high Z scores surrounded by other bins with high Z scores), while negative Z scores indicate low values of bins surrounded by other low values of bins (i.e., a cold spot) (Getis and Ord 2010). A Gi* near 0 indicates a random distribution. A Mann-Kendall test is a non-parametric statistical method that calculates a ranking analysis of the number and value of each time interval and time order (Mann 1945, Kendall 1948). The test calculates values for each period in the test to derive a time-series spot pattern for each bin. The results (cluster from Getis-Ord Gi* and trend from Mann-Kendall) are then combined and categorized as new hot/cold spots, consecutive hot/cold spots, intensifying hot/cold spots, persistent hot/cold spots, diminishing hot/cold spots, oscillating hot/cold spot, historical hot/cold spot, and no detectable pattern.

Input parameters for the Emerging Hot Spot Analysis tool include: 1) time step; 2) conceptualization of spatial relationships; and 3) polygon analysis mask. The time step determines which periods are analyzed together. Given that MVCs are considered rare and random events with varying frequencies over time, the Highway Safety Manual recommends three to five years for analyses (Manual and Manual 2010). We selected 3 years as the time step, meaning five Getis-Ord Gi* results were compared in the analysis. We then decided on a conceptualization of spatial relationships. These parameters determined which bins should be considered as part of the same neighborhood. We conducted exploratory analyses and decided on the fixed distance band as the most appropriate for outcomes along the road network. A fixed distance band conceptualization means that we determine the sphere of influence or the area in which features are geographically related (the neighborhood). We selected this to evaluate statistical properties at a fixed spatial scale. In this conceptualization, neighboring bins within the distance threshold influence computations on the bin, whereas bins outside the distance do not have an influence on a bin’s computations. *Figure 2* demonstrates the concept of a space-time cube (including the geographic components of latitude and longitude and the time component). Labels are provided for bins (in our case two km x two km bins) and neighborhoods (the area in which features are related).

*Figure 2 Space-time cube visualization, adapted from ESRI (ESRI)*

*
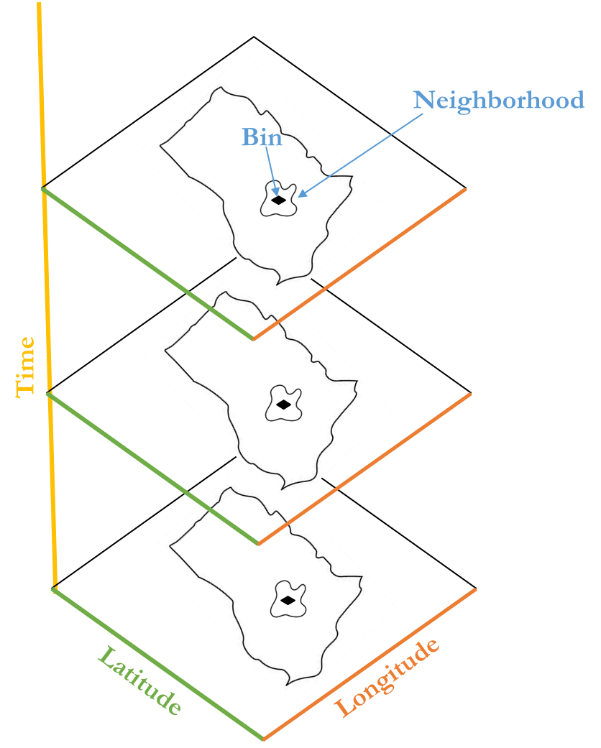
*

To determine the most appropriate distance, we used the methods nearest neighbor analysis and an incremental spatial autocorrelation, for every three years for both urban and rural areas. Results indicated that the average 8-neighbor distance, recommended as a minimum for spatial analysis, was about five km for rural and two km for urban areas, with minor variations in the periods. The Global Moran’s I result indicated that significant spatial autocorrelation began at about 500 meters (*Supplementary Materials).* We selected two km as the distance for urban areas and eight km as the distance for rural areas. According to geospatial experts, selecting an appropriate neighborhood based on subjective judgment from a range of density surfaces is appropriate (Atkinson and Unwin 2002, Brimicombe 2009). Experts also note that such decisions should be related to context of the study (i.e., relevant for road safety in Ghana)(Brimicombe 2009)

Although there were some variations in the results based on neighborhood distances (i.e., more or fewer types of outcomes), trends of clusters were consistent. For the third parameter, polygon analysis mask, we implemented a stratified analysis as most MVCs took place in dense, urban areas, whereas we sought to understand spatiotemporal associations in both urban and rural areas. Thus, we compared urban bins to urban bins and rural bins to rural bins with differing distance thresholds.

Outcome definitions for emerging hot spot analysis tool in ArcGIS Pro

In the following table, we present the pre-defined pattern types and definitions for the emerging hot spot analysis tool in ArcGIS Pro (198).

| **Pattern type** | **Definition** |
| --- | --- |
| New hotspot | A location that is a statistically significant hotspot for the final time step and has never been a statistically significant hotspot before. |
| Consecutive hotspot | A location with a single uninterrupted run of at least two statistically significant hotspot bins in the final time-step intervals. The location has never been a statistically significant hotspot prior to the final hotspot run and less than 90 percent of all bins are statistically significant hotspots. |
| Intensifying hotspot | A location that has been a statistically significant hotspot for 90 percent of the time-step intervals, including the final time step. In addition, the intensity of clustering of high counts in each time step is increasing overall and that increase is statistically significant. |
| Persistent hotspot | A location that has been a statistically significant hotspot for 90 percent of the time-step intervals with no discernible trend in the intensity of clustering over time. |
| Diminishing hotspot | A location that has been a statistically significant hotspot for 90 percent of the time-step intervals, including the final time step. In addition, the intensity of clustering in each time step is decreasing overall and that decrease is statistically significant. |
| Sporadic hotspot | A statistically significant hotspot for the final time-step interval with a history of also being an on-again and off-again hotspot. Less than 90 percent of the time-step intervals have been statistically significant hotspots and none of the time-step intervals have been statistically significant cold spots |
| Oscillating hotspot | A statistically significant hotspot for the final time-step interval that has a history of also being a statistically significant cold spot during a prior time step. Less than 90 percent of the time-step intervals have been statistically significant hotspots. |
| Historical hotspot | The most recent time period is not hot, but at least 90 percent of the time-step intervals have been statistically significant hotspots. |
| New cold spot | A location that is a statistically significant cold spot for the final time step and has never been a statistically significant cold spot before. |
| Consecutive cold spot | A location with a single uninterrupted run of at least two statistically significant cold spot bins in the final time-step intervals. The location has never been a statistically significant cold spot prior to the final cold spot run and less than 90 percent of all bins are statistically significant cold spots. |
| Intensifying cold spot | A location that has been a statistically significant cold spot for 90 percent of the time-step intervals, including the final time step. In addition, the intensity of clustering of low counts in each time step is increasing overall and that increase is statistically significant. |
| Persistent cold spot | A location that has been a statistically significant cold spot for 90 percent of the time-step intervals with no discernible trend in the intensity of clustering of counts over time. |
| Diminishing cold spot | A location that has been a statistically significant cold spot for 90 percent of the time-step intervals, including the final time step. In addition, the intensity of clustering of low counts in each time step is decreasing overall and that decrease is statistically significant. |
| Sporadic cold spot | A statistically significant cold spot for the final time-step interval with a history of also being an on-again and off-again cold spot. Less than 90 percent of the time-step intervals have been statistically significant cold spots and none of the time-step intervals have been statistically significant hotspots. |
| Oscillating cold spot | A statistically significant cold spot for the final time-step interval that has a history of also being a statistically significant hotspot during a prior time step. Less than 90 percent of the time-step intervals have been statistically significant cold spots. |
| Historical cold spot | The most recent time is not cold, but at least 90 percent of the time-step intervals have been statistically significant cold spots. |
| No detectable pattern | Does not fall into any of the hotspot patterns defined below. |

Additional information on calculating appropriate distance bands

In the following table, we present results from the “calculate distance band from neighbor count” tool in ArcGIS Pro. This determines the distance to the closest eight neighbors and was used to inform our neighborhood designation for the emerging hot spot analysis.

‘*Calculate distance band from neighbor count’ results (Eight neighbors)*

| **3-year period** | **Type of area** | **Average** | **Maximum** |
| --- | --- | --- | --- |
| 2006-2008 | Rural | 4158.83 | 67977.36 |
| 2006-2008 | Urban | 2137.75 | 88061.98 |
| 2009-2011 | Rural | 3769.39 | 60598.66 |
| 2009-2011 | Urban | 2047.36 | 88136.43 |
| 2012-2014 | Rural | 4520.97 | 50970.82 |
| 2012-2014 | Urban | 2376.72 | 93841.96 |
| 2015-2017 | Rural | 5515.24 | 58322.01 |
| 2015-2017 | Urban | 3709.09 | 86875.77 |
| 2018-2020 | Rural | 4706.42 | 52603.80 |
| 2018-2020 | Urban | 2879.49 | 87856.40 |

We also conducted incremental spatial autocorrelation tests for each urban and rural area for each three-year period. We present an example of those results for 2006 to 2008 in rural areas below.

*Incremental spatial autocorrelation results – rural areas 2006-2008*

*
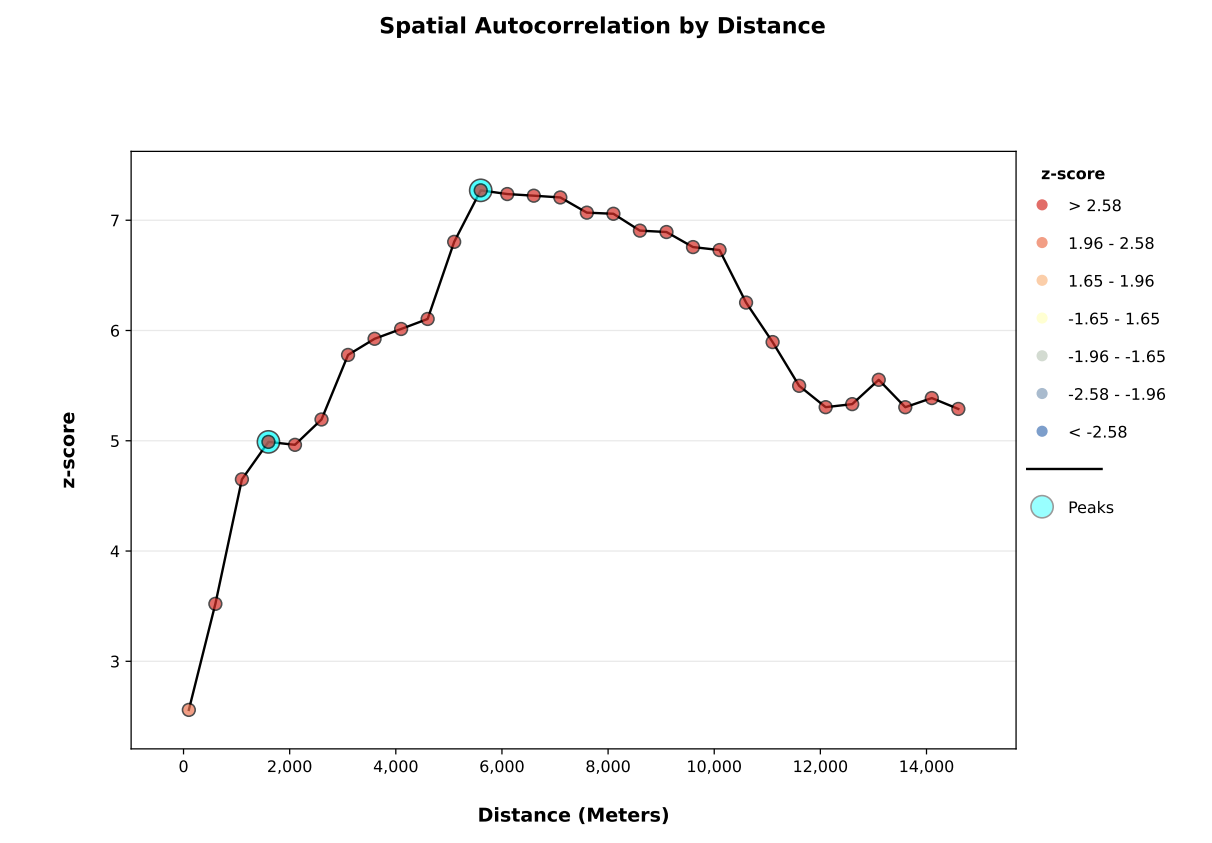
*

Distribution of injury severity index from 2005 to 2020

We presented the mean of the injury severity index in the main text. In an effort to understand the potential changes in the distribution, we plotted a histogram for each year, shown below.

*Histograms of road traffic injury severity index - 2005 to 2020*


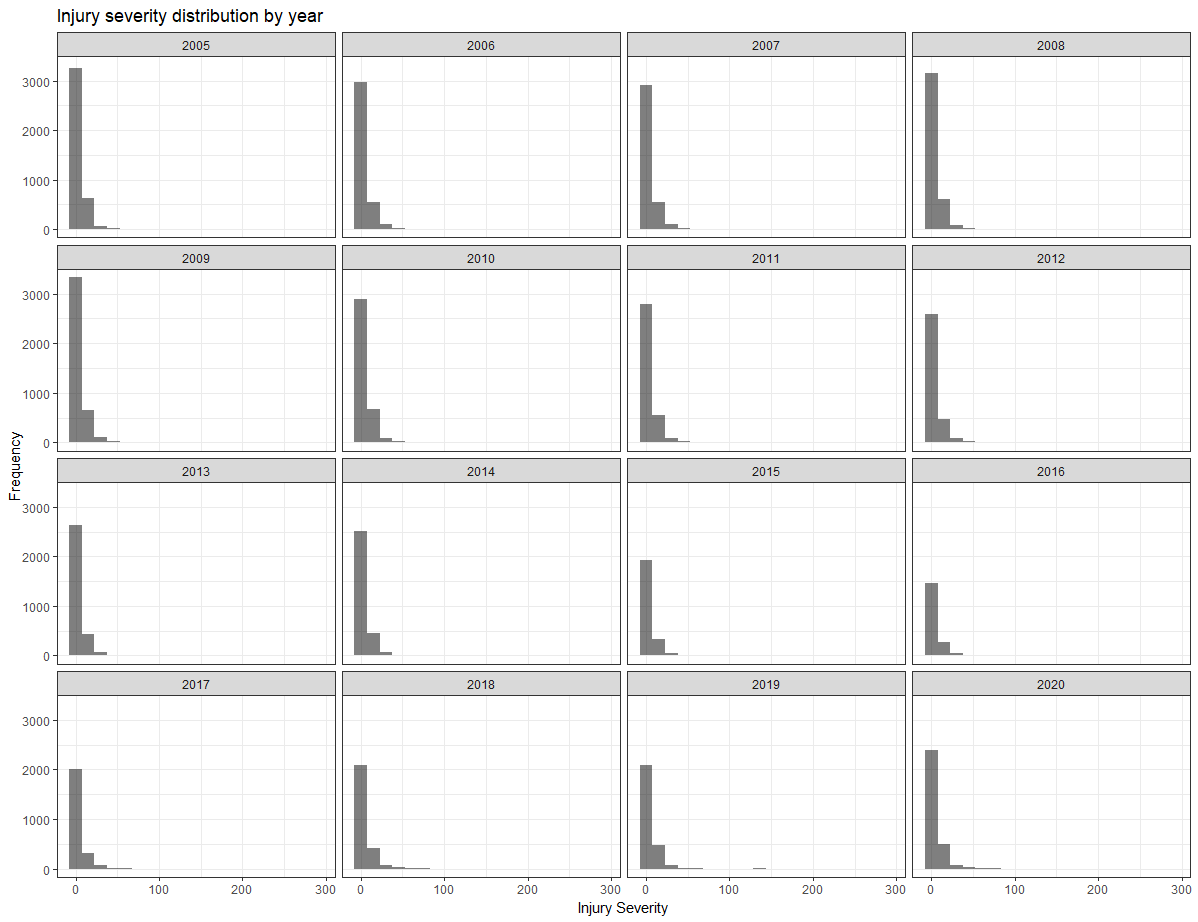


National maps of rural and urban spatiotemporal clusters for by outcome

*Emerging hot spot results: injury severity index (2005-2020)*


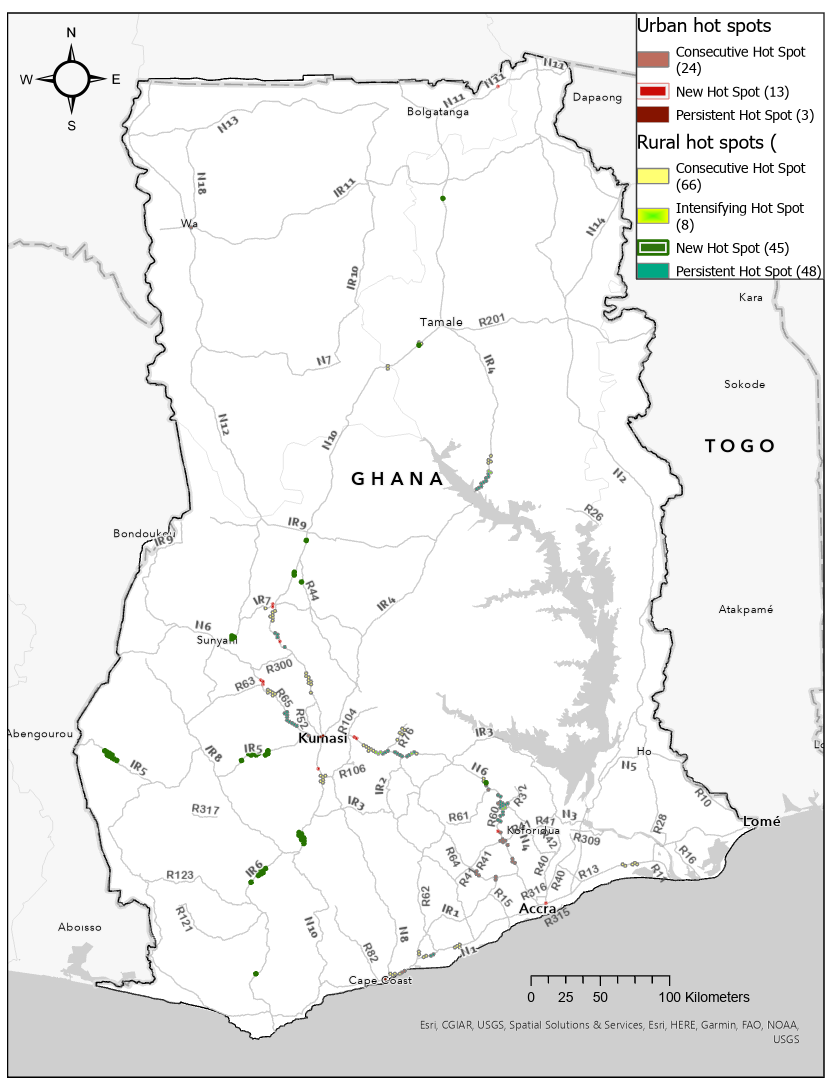


*Emerging hot spot results – injury count, 2005 to 2020*


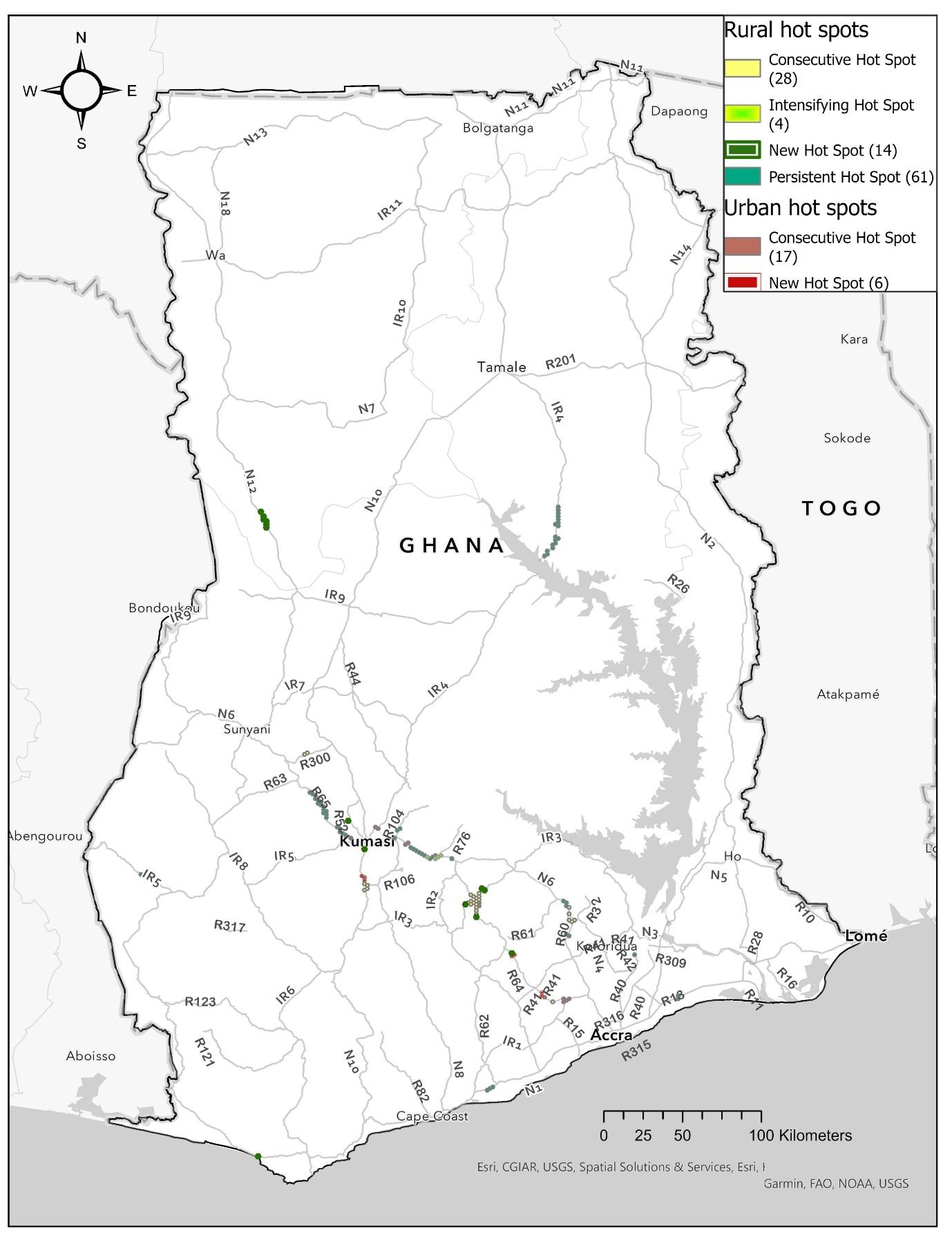


*Emerging hot spot results – severe injury count, 2005 to 2020*


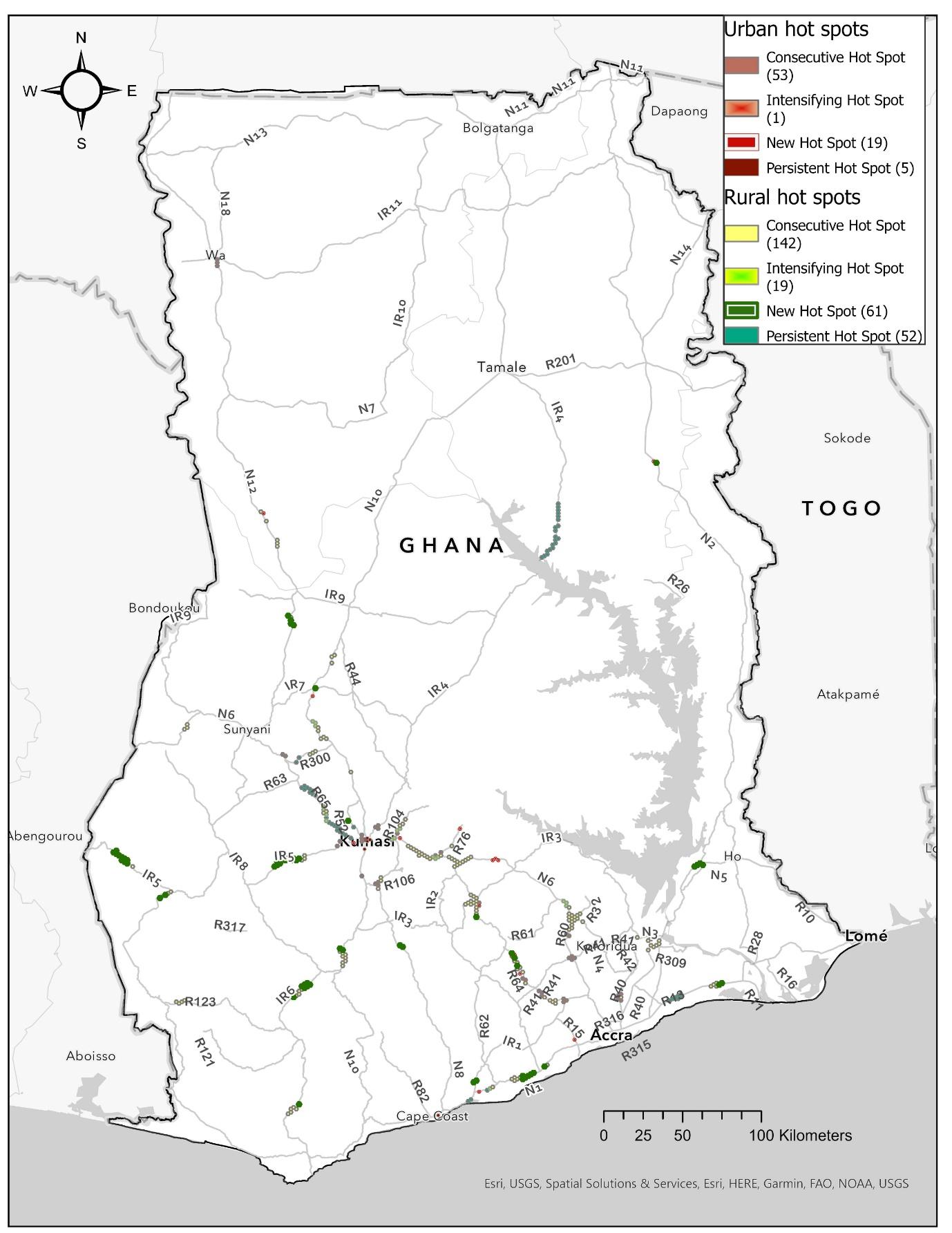


*Emerging hot spot results – death count, 2005 to 2020*


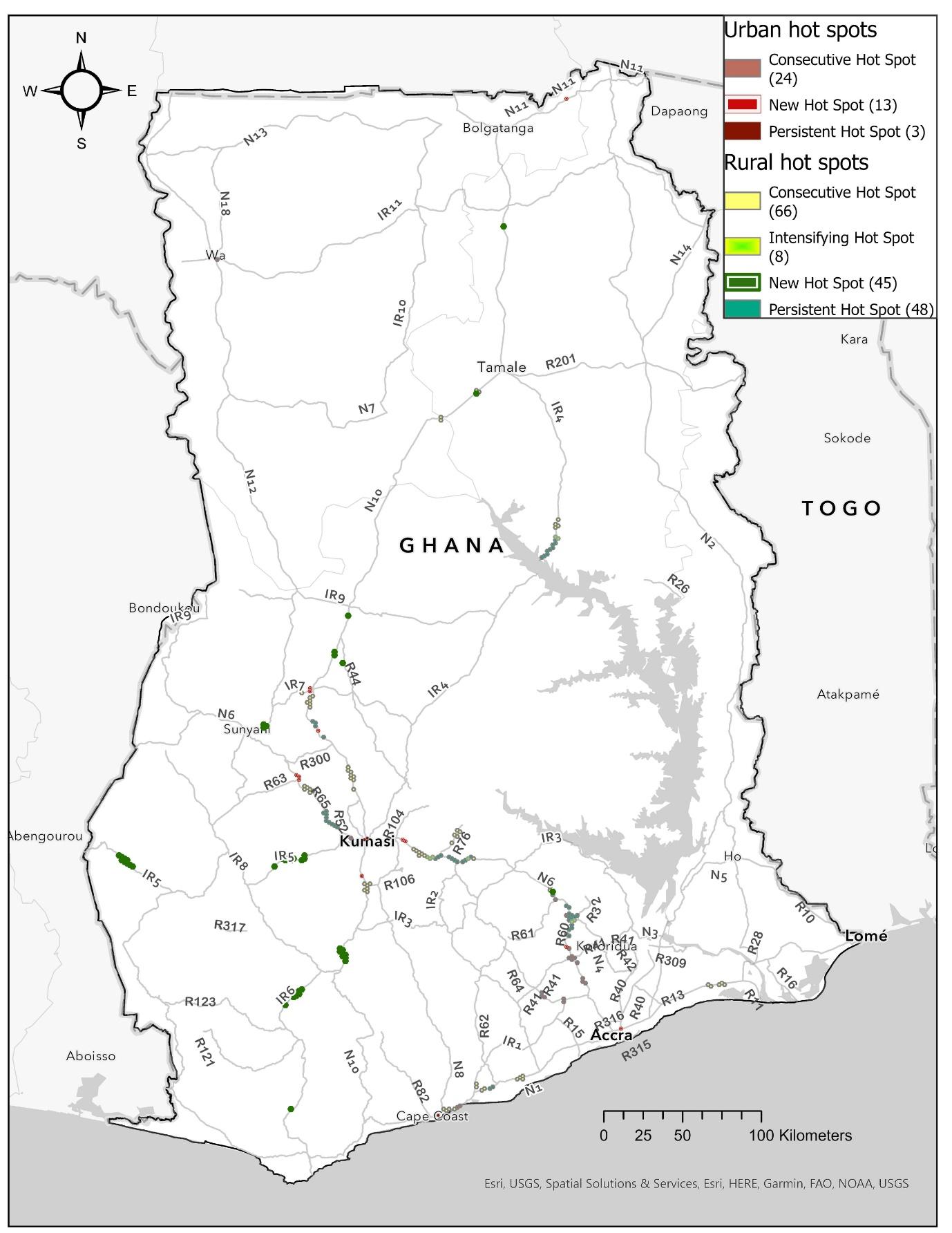

Supplement: Supplementary file 1 — Supplementary Material 1 [file 12889_2024_18915_MOESM1_ESM.docx]
